# Mediation effect of cognitive impairment for the relationship of type 2 diabetes mellitus with mortality among elderly individuals

**DOI:** 10.3389/fendo.2024.1392326

**Published:** 2024-06-03

**Authors:** Boyang Wei, Jun He

**Affiliations:** Nutrition Department, The 960th Hospital of the Chinese People’s Liberation Army, Jinan, Shandong, China

**Keywords:** mediation effect, cognitive impairment, type 2 diabetes mellitus, all-cause mortality, National Health and Nutrition Examination Survey

## Abstract

**Objectives:**

To investigate the potential mediating role of cognitive impairment on the link between type 2 diabetes mellitus (T2DM) and mortality among elderly individuals using data from the National Health and Nutrition Examination Survey (NHANES) database.

**Methods:**

Totally, 1,891 individuals from the NHANES database were included in this cohort study. All-cause mortality was considered study endpoint. Cognitive impairment was assessed by digit symbol substitution test (DSST). Adopted weighted logistic regression analyses to explore the relationship of T2DM with cognitive impairment. Constructed weighted Cox proportional hazard models to investigate the relationship of T2DM with all-cause mortality. We employed distribution-of-the-product method to investigate the mediating effect. RMediation software package was used to calculate the 95% confidence interval (CI) of the distribution-of-the-product. If CI does not contain 0, it suggests a significant mediation effect.

**Results:**

The findings from the weighted logistic regression revealed that individuals with T2DM had a significantly higher likelihood of experiencing cognitive impairment [odds ratio =1.86, 95% CI: 1.39–2.49]. The result showed that T2DM was related to an increased all-cause mortality (hazard ratio=1.37, 95%CI: 1.01–1.87). Importantly, the mediation effect of cognitive impairment on the relationship of T2DM with all-cause mortality is significant (95%CI: 0.06–0.59). The percentage of mediation effect was calculated as 16.2%.

**Conclusion:**

Our study suggested that the presence of cognitive impairment plays a significant role in explaining the link between T2DM and all-cause mortality in older individuals.

## Introduction

Diabetes mellitus is a prevalent metabolic disorder (1, 2). According to the World Health Organization (WHO), there is an anticipated rise in the prevalence of diabetes among adults, with type 2 diabetes mellitus (T2DM) accounting for the majority of cases (3).

Patients diagnosed with type 2 diabetes mellitus (T2DM) who are in the older age group face an increased macrovascular and microvascular complications through different pathogenetic pathways, ultimately resulting in a heightened mortality risk in these patients (4, 5). The mortality rate is approximately 34.11%, resulting in a significant disease burden (6). Therefore, identification of potential risk factors in older individuals with T2DM can help to reduce mortality.

Cognitive impairment is frequently observed among the elderly population and seriously threaten their health (7). T2DM is believed to be linked to cognitive decline (8). The metabolic imbalance induced by chronic hyperglycemia and hyperinsulinemia may facilitate oxidative stress, inflammation, and endothelial impairment, thereby accelerating cerebrovascular atherosclerosis and neurodegenerative damage, and further exacerbating cognitive dysfunction (9).

Cognitive function decreased with the increase of hemoglobin A1c (HbA1c) (10, 11). In a study conducted by Li w, et al., it was found that elderly Chinese individuals with T2DM had an increased susceptibility to developing Alzheimer’s disease after adjusting for possible confounders (12). In addition, several studies have also pointed out that older adults with lower cognitive function face a higher risk of all-cause death compared to the general older adults (13, 14). An epidemiological analysis in 2,977 middle-aged and older individuals with T2DM showed that for patients with T2DM, the cognitive function score, as measured by the digit symbol substitution test (DSST), was linked with the risk of all-cause mortality (15). These findings also indicated that the assessment of adverse outcomes in patients with T2DM should take into account the significance of cognitive impairment. Nowadays, the utilization of mediation analysis in clinical research has witnessed a growing trend (16), which can decompose the total effect of a variable into direct and indirect effects, helping to consider the effectiveness of intervention strategies (17, 18). However, limited research has been conducted on the mediating role of cognitive impairment in the association of T2DM with mortality in older populations.

Our study aimed at exploring the potential role of cognitive dysfunction as a mediator in the link between T2DM and mortality among elderly individuals. This could serve as a valuable guideline for actively screening older patients with T2DM for cognitive impairment, aiming to mitigate the likelihood of mortality.

## Methods

### Study population

This study utilized data from the National Health and Nutrition Examination Survey (NHANES) database to conduct a retrospective cohort analysis. NHANES is a stratified, multistage probability survey of United States population (19), which examines nationally representative sample of approximately 5,000 people each year. All participants were required to complete household interviews, laboratory measurements, and physical examinations (https://www.cdc.gov/nchs/nhanes/about_nhanes.htm). NHANES survey procedures and protocols received approved from the Research Ethics Review Board of the National Center for Health Statistics, and all participants provided written consent after being informed. This study adhered to the Helsinki Declaration.

**Figure 1:**
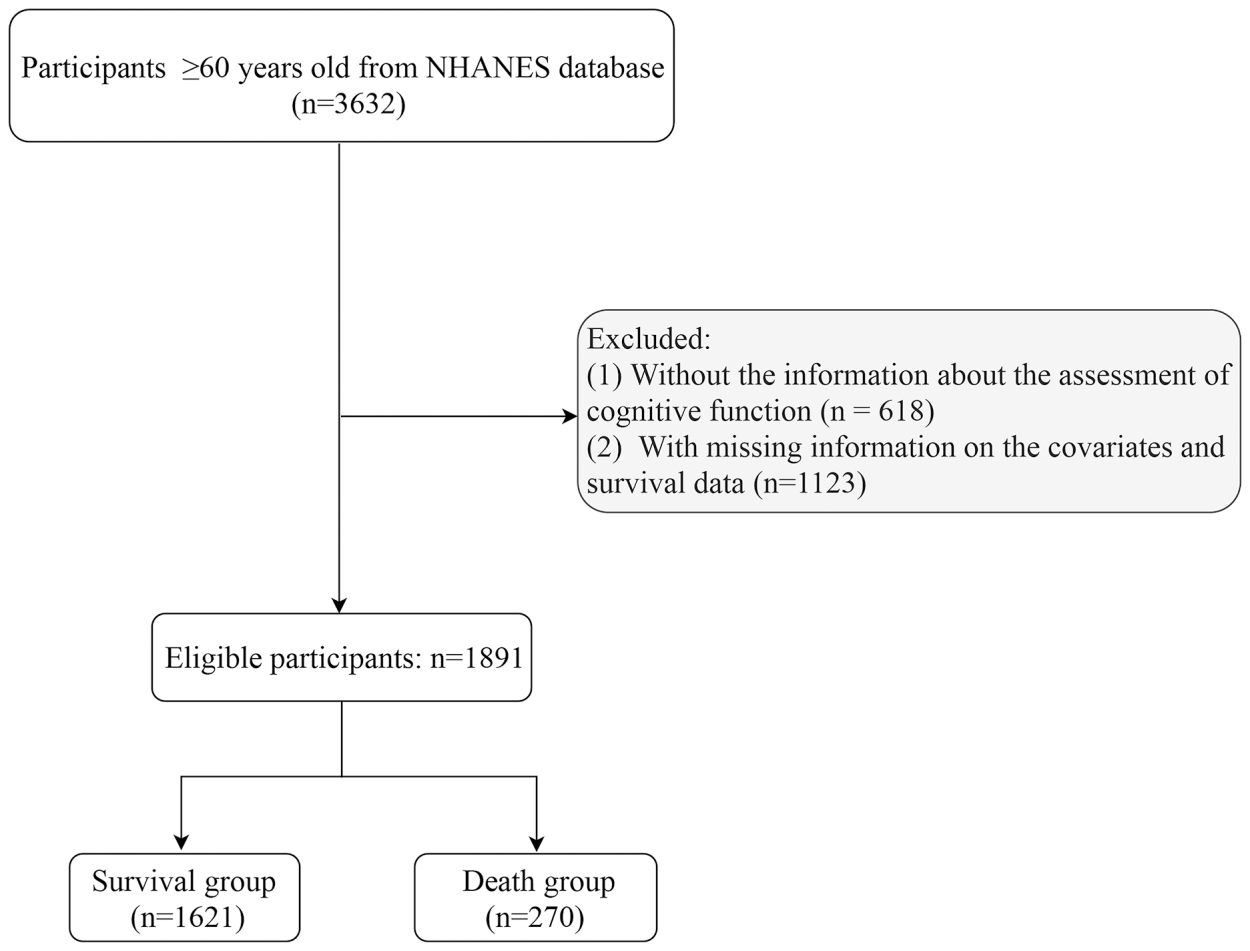
The screening process of participants.

Subjects were included from NHANES database 2011–2014. Inclusion criteria: being at least 60 years of age. Exclusion criteria: (1) missing information about the assessment of cognitive function and T2DM; (2) missing information on survival data and key covariates [including height, weight, history of cardiovascular disease (CVD), physical activity, and depression] (**Figure 1**).

### Data collection

#### Identification of type 2 diabetes patients

T2DM was considered an exposure factor in this study. It was defined as HbAlc ≥6.5% or fasting blood glucose ≥7.0 mmol/L or self-reported T2DM (participants who answered yes to the following questions were defined as having T2D: “Have you ever been told by a doctor or health professional that you have diabetes or sugar diabetes”) or taking diabetes drugs.

#### Assessment of cognitive impairment

In the NHANES database, individuals aged 60 years or older were asked to perform cognitive function assessment (https://wwwn.cdc.gov/Nchs/Nhanes/2013–2014/CFQ_H.htm). DSST is a widely recognized evaluation of cognitive abilities that evaluates executive functioning and the speed at which information is processed. It has been extensively utilized in assessing cognitive function among participants aged ≥60 years from NHANES database (20). DSST was used to measure the ability of processing speed. The examination was conducted utilizing a paper document featuring a header consisting of nine numerical figures matched with corresponding symbols. Individuals were instructed to draw symbols under the corresponding numbers in 133 boxes adjacent to the numbers for two minutes. The cumulative score for DSST is determined by accurately matching a total of 133 items (21). Since there is no gold standard for a threshold score for a DSST representing cognitive impairment, we have identified the lowest quartile in the study population (DSST ≤36) as cognitive impairment in this analysis (20).

#### Outcome variable

All-cause mortality was considered study endpoint. In NHANES database, the status of death and the time of follow-up were extracted through the public-use linked mortality file obtained from the NCHS (https://www.cdc.gov/nchs/data-linkage/mortality.htm#print) and matched with the ID of participants from the NHANES database. The duration of follow-up for the study participants had a median value of 79.78 [68.92, 94.34] months.

#### Possible covariates

Data of the participants were collected: age (years), race, gender, marital status, body mass index (BMI, kg/m^2^), poverty-to-income ratio (PIR), drinking alcohol, smoking, diastolic blood pressure (DBP, mmHg), systolic blood pressure (SBP, mmHg), physical activity, history of CVD, dyslipidemia, and hypertension, cancer, estimated glomerular filtration rate (eGFR, mL/min/1.73m^2^), creatinine (mg/dL), Mediterranean Diet (MED), depression.

A metabolic equivalent (MET) was employed to characterize energy consumption for specific activity (22). In this study, physical activity was calculated as energy consumption, where energy consumption (MET·min) is determined by multiplying MET with exercise time for the corresponding activity (min). The eGFR_CKD-EPI_ equation from the chronic kidney disease epidemiology collaboration was utilized to calculate the eGFR. eGFR_CKD-EPI_ = 141×min (Scr/κ, 1) ^α^×max (Scr/κ, 1) ^-1.029^×0.993 ^age^×1.108 (if female) (23). Coefficient κ is 0.7 for female population and 0.9 for male population, while α is -0.329 for females and -0.411 for males. The term “min” indicates the minimum of Scr/κ or 1, whereas “max” indicates the maximum of Scr/κ or 1. The abbreviation “Scr” stands for serum creatinine (mg/dL).

Patient Health Questionnaire-9 (PHQ-9) was utilized to evaluate depression (24). The total score ranges from 0–27. A PHQ-9 score ≥ 10 represented clinically significant depression.

Adherence to MED scores was calculated based on the consumption of nine components (25), including vegetables, legumes, cereals, fruit and nuts, fish and seafood, meat and meat products, dairy products, the ratio of monounsaturated to saturated fats, and alcohol. A score of 1 was assigned to people whose consumption of presumed beneficial foods (whole grains, vegetables, fruit, nuts, legumes, fish, and the ratio of monounsaturated fatty acids-to-saturated fatty acids) exceeded the median and whose consumption of presumed detrimental foods (red and processed meat) fell below the median. Conversely, a score of 0 was assigned to all others. In terms of alcohol, a score of 1 was given to males who had a daily consumption ranging from 10 to 25g, and females who consumed between 5 and 15g per day.

### Statistical analysis

Continuous variables were described by Mean (standard error) (SE), and an independent sample t test was conducted for groups comparisons. Categorical variables were described by the number of cases and composition ratio n (%), with Chi-square employed for between-group comparisons.

Weighted logistic regression models were established to explore the potential link between T2DM and cognitive impairment, with cognitive impairment as outcome variable, and odds ratio (OR) values with 95% confidence interval (CI) were presented. The crude model did not account for confounders (Model 1). Model 2 was adjusted for age, gender, and race. Model 3 was further adjusted for age, gender, race, physical activity, history of CVD, hypertension, cancer, and dyslipidemia by stepwise regression. Additionally, a dispersion model correlated with DSST and blood glucose was also developed (26). Weighted Cox proportional hazard models were utilized to examine the relationship between T2DM and all-cause mortality, using all-cause mortality as the outcome variable. Hazard ratios (HRs) and 95% CIs were calculated. We employed distribution-of-the-product method to investigate how cognitive impairment mediates the link between T2DM and mortality (27). The RMediation software package allows for the acquisition of a 95%CI pertaining to the distribution-of-the-product. If the 95%CI does not contain 0, it indicates that there is a significant mediation effect (28). Simultaneously, if there was no interaction between T2DM and DSST score on all-cause mortality, the mediated proportion was determined by comparing the total effect with the controlled direct effect (26). The percentage of mediation effect was calculated using the following equation: (HR^I^ - HR^II^)/(HR^I^-1) × 100, where HR^I;^ represented the HR in Model I and HR ^I^ denoted the HR in Model II. Multiple interpolation method was employed to fill in the missing variables, and a sensitivity analysis was conducted on both the pre- and post-interpolation data (see **Supplementary Table 1**).

Utilizing Python 3.9 for data cleaning and missing value processing, SAS 9.4 for model statistical analysis, and R 4.0.3 software for mediation effect analysis. A significance level of less than 0.05 was deemed statistically significant.

## Results

### Baseline characteristics

Data from 3,632 participants were extracted from the NHANES database from 2011–2014. After excluding participants with missing information about cognitive function assessment (n=618), and those with missing information on covariates and survival data (n=1123), 1,891 participants were involved (**Figure 1**). These participants were then categorized into survival group (n=1621) and non-survival group (n=270). The all-cause mortality rate was 14.28%. Of these 1891 participants, 519 (21.14%) had T2DM, and 480 (12.75%) had cognitive impairment. The mean age was 68.57 years, including 987 (48.43%) males and 904 (51.57%) females. More detailed characteristics were presented in **Table 1**. In addition, notable variances were observed in terms of age, gender, marital status, PIR, smoking, DBP, history of CVD and hypertension, cancer, physical activity, creatinine, and eGFR between survival and non-survival groups.

**Table 1:** Baseline characteristics of participants.

Variables	Total (n=1891)	Survival group (n=1621)	Non-survival group (n=270)	Statistics	*P*
Age, year, Mean (S.E)	68.57 (0.22)	67.74 (0.20)	74.45 (0.50)	t=-13.17	<0.001
Gender, n (%)				χ^2^=5.65	0.017
Male	987 (48.43)	818 (47.27)	169 (56.68)		
Female	904 (51.57)	803 (52.73)	101 (43.32)		
Race, n (%)				χ^2^=5.10	0.078
Non-Hispanic White	883 (79.89)	719 (79.33)	164 (83.90)		
Non-Hispanic Black	443 (7.95)	385 (7.97)	58 (7.85)		
Other	565 (12.15)	517 (12.70)	48 (8.25)		
Marital status, n (%)				χ^2^=9.34	0.009
Married	1077 (64.55)	946 (66.01)	131 (54.15)		
Never married	105 (3.94)	89 (3.77)	16 (5.08)		
Other	709 (31.51)	586 (30.21)	123 (40.78)		
BMI, kg/m^2^, Mean (S.E)	28.37 (0.20)	28.44 (0.19)	27.88 (0.43)	t=1.54	0.134
PIR, Mean (S.E)	3.26 (0.08)	3.34 (0.09)	2.74 (0.13)	t=4.16	<0.001
Smoking, n (%)				χ^2^=7.33	0.007
No	936 (49.49)	826 (50.75)	110 (40.51)		
Yes	955 (50.51)	795 (49.25)	160 (59.49)		
Drinking alcohol, n (%)				χ^2^=0.37	0.545
No	568 (24.40)	491 (24.13)	77 (26.34)		
Yes	1323 (75.60)	1130 (75.87)	193 (73.66)		
SBP, mmHg, Mean (S.E)	130.73 (0.48)	130.34 (0.44)	133.52 (1.81)	t=-1.80	0.082
DBP, mmHg, Mean (S.E)	69.00 (0.61)	69.58 (0.63)	64.87 (0.98)	t=5.45	<0.001
Physical activity, n (%)				χ^2^=9.57	0.002
Physical activity > 360 MET· min	906 (52.02)	805 (53.40)	101 (42.17)		
Physical activity360 MET· min	985 (47.98)	816 (46.60)	169 (57.83)		
History of CVD, n (%)				χ^2^=17.50	<0.001
No	1541 (81.81)	1354 (83.63)	187 (68.87)		
Yes	350 (18.19)	267 (16.37)	83 (31.13)		
History of hypertension, n (%)				χ^2^=12.79	<0.001
No	598 (36.23)	539 (38.08)	59 (23.05)		
Yes	1293 (63.77)	1082 (61.92)	211 (76.95)		
History of dyslipidemia, n (%)				χ^2^=2.83	0.092
No	312 (15.13)	258 (14.30)	54 (21.02)		
Yes	1579 (84.87)	1363 (85.70)	216 (78.98)		
History of cancer, n (%)				χ^2^=4.41	0.036
No	1515 (76.08)	1322 (77.06)	193 (69.06)		
Yes	376 (23.92)	299 (22.94)	77 (30.94)		
eGFR, mL/min/1.73m^2^, Mean (S.E)	83.22 (0.50)	84.36 (0.50)	75.12 (1.30)	t=7.18	<0.001
Creatinine, mg/dL, Mean (S.E)	0.96 (0.01)	0.94 (0.01)	1.10 (0.04)	t=-4.30	<0.001
MED, score, Mean (S.E)	4.70 (0.12)	4.71 (0.13)	4.62 (0.18)	t=0.38	0.710
Depression, n (%)				χ^2^=1.06	0.303
PHQ-9 scores < 10	1756 (94.66)	1504 (94.48)	252 (95.94)		
PHQ-9 scores ≥ 10	135 (5.34)	117 (5.52)	18 (4.06)		
T2DM, n (%)				χ^2^=7.59	0.006
No	1372 (78.86)	1193 (79.68)	179 (73.06)		
Yes	519 (21.14)	428 (20.32)	91 (26.94)		
DSST score, n (%)				χ^2^=28.38	<0.001
DSST score >36	1411 (87.25)	1250 (89.11)	161 (73.98)		
DSST score36	480 (12.75)	371 (10.89)	109 (26.02)		
Time, month, Mean (S.E)	80.65 (1.23)	84.40 (1.26)	53.92 (2.10)	t=13.33	<0.001

BMI, body mass index; PIR, poverty-to-income ratio; SBP, systolic blood pressure; DBP, diastolic blood pressure; CVD, cardiovascular disease; eGFR, estimate glomerular filtration rate; MED, Mediterranean Diet; MET, metabolic equivalent; PHQ-9, Patient Health Questionnaire-9; T2DM, type 2 diabetes mellitus; DSST, digit symbol substitution test; “P” indicated the results of difference analysis between survival group and death group.

### Association of T2DM with cognitive impairment

Possible confounding factors that may influence all-cause mortality were assessed using weighted univariate Cox proportional hazard analysis (**Supplementary Table 2**). A weighted logistic regression was then performed. As shown in **Table 2**, all three models conducted in this study indicated that there exists a significant association between T2DM and cognitive impairment (Model 1: OR=2.54, 95%CI: 2.00–3.22; Model 2: OR=1.95, 95%CI: 1.49–2.56; Model 3: OR=1.86, 95%CI: 1.39–2.49). Furthermore, in this analysis, 519 participants had T2DM. The mean (SE) fasting glucose of participants with type 2 diabetes was 143.67 (4.45) mg/dL and the mean HbA1c was 6.94% (0.10). Of the 519 patients diagnosed with T2DM, a total of 339 exhibited cognitive impairment. The mean (SE) fasting glucose among these individuals was recorded as 142.28 (5.02) mg/dL, while their mean HbA1c value stood at 6.88% (0.10). Among the remaining 180 T2DM patients without cognitive impairment, the mean (SE) fasting glucose was measured at 148.03 (5.99) mg/dL and their mean HbA1c value was found to be 7.18% (0.20). As shown in **Supplementary Figure 1**, **Supplementary Figure 2**, DSST was negatively correlated with fasting glucose (r=-0.18) and HbA1c (r= -0.19), respectively.

**Table 2:** The association of T2DM and cognitive impairment.

Variables	Model 1		Model 2		Model 3	
	OR (95% CI)	*P*	OR (95% CI)	*P*	OR (95% CI)	*P*
T2DM						
No	Ref		Ref		Ref	
Yes	2.54 (2.00-3.22)	<0.001	1.95 (1.49-2.56)	<0.001	1.86 (1.39-2.49)	<0.001

T2DM, type 2 diabetes mellitus; OR, odds ratio; CI, confident interval; Ref, reference.

Model 1 did not adjust for confounders.

Model 2 adjusted for age, gender, and race.

Model 3 adjusted for age, gender, race, physical activity, history of cardiovascular disease, hypertension, cancer, and dyslipidemia.

### Mediating role of cognitive impairment

**Figure 2:**
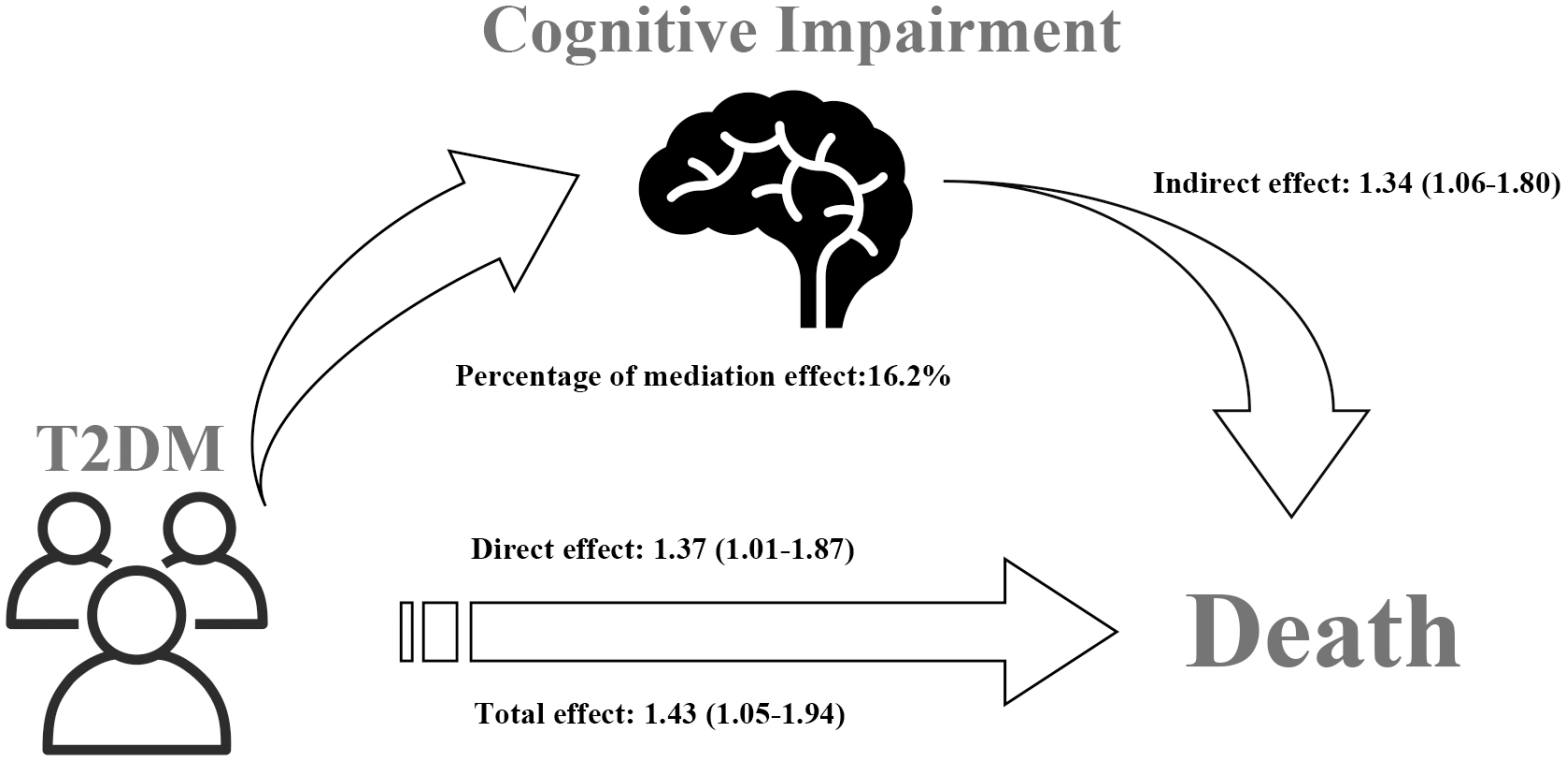
Mediation effect of cognitive impairment on the association between T2DM and all-cause mortality.

The correlation of T2DM with all-cause mortality is presented in **Table 3**. After accounting for age, gender, race, physical activity, history of CVD, hypertension, cancer, and dyslipidemia, Model I (HR=1.43, 95%CI: 1.05–1.94) revealed that T2DM was related to an elevated risk of all-cause mortality. This finding was consistent in Model II as well (HR=1.37, 95%CI: 1.01–1.87). Importantly, the study examined the mediating role of cognitive impairment on the relationship T2DM and all-cause mortality. The CI of distribution-of-the-product did not include zero (95%CI: 0.06–0.59), indicating a significant mediation effect. The total effect of T2DM on all-cause mortality was found to be 1.43 (95%CI: 1.05–1.94), while the direct effect of T2DM on all-cause mortality risk was estimated at 1.37 (95%CI: 1.01–1.87). The indirect effect of T2DM on all-cause mortality risk was found to be 1.34 (95%CI: 1.06–1.80). As shown in **Table 4**, there was no interaction observed between cognitive impairment and T2DM in relation to all-cause mortality. The percentage of mediation effect were based on the comparison of total effects with controlled direct effects. By calculation, the percentage of mediation effect was 16.2% (**Table 3**). **Figure 2** shows mediation effect of cognitive impairment for the association of T2DM with all-cause mortality.

**Table 3:** The mediating role of cognitive impairment for the association between T2DM and all-cause mortality.

Variables	Crude Model	Model I	Model II	CI of distribution of the product (95% CI)	Indirect effect HR (95% CI)	Percentage of mediation effect
	HR (95% CI)	HR ^I^ (95% CI)	HR ^II^ (95% CI)			
T2DM						
No	Ref	Ref	Ref			
Yes	1.45 (1.10-1.93)	1.43 (1.05-1.94)	1.37 (1.01-1.87)	0.29 (0.06-0.59)	1.34 (1.06-1.80)	16.2%

T2DM, type 2 diabetes mellitus; HR, hazard ratio; CI, confident interval; Ref, reference.

Crude Model: did not adjust for confounders.

Model I adjusted for age, gender, race, physical activity, history of cardiovascular disease, hypertension, cancer, and dyslipidemia.

Model II adjusted for age, gender, race, physical activity, history of cardiovascular disease, hypertension, cancer, and dyslipidemia, and DSST score.

**Table 4:** The interaction between T2DM and DSST score on all-cause mortality.

Interaction item	Model 1		Model 2		Model 3	
	HR (95% CI)	*P*	HR (95% CI)	*P*	HR (95% CI)	*P*
T2DM and DSST score	0.54 (0.32-0.94)	0.029	0.68 (0.42-1.10)	0.115	0.70 (0.44-1.10)	0.118

T2DM, type 2 diabetes mellitus; HR, hazard ratio; CI, confident interval; DSST, digit symbol substitution test.

Model 1 did not adjust for confounders.

Model 2 adjusted for age, gender, and race.

Model 3 adjusted for age, gender, race, physical activity, and history of cardiovascular disease, hypertension, and dyslipidemia.

## Discussion

Notably, this research presented findings indicating that cognitive impairment played mediating role in the link between T2DM and all-cause mortality among elderly individuals from the NHANES database. In addition, we found that cognitive impairment mediated 14% of the link between T2DM and all-cause mortality, as determined through mediation analysis. These results indicated that cognitive impairment has a significant influence on all-cause mortality of patients with T2DM. Therefore, it is crucial to give consideration to the development of cognitive impairment in individuals with T2DM as a preventive measure against elevated mortality rates among T2DM patients.

T2DM poses a significant health concern among the older adults, bringing higher mortality. A study conducted over a period of 18 years reported that individuals diagnosed with T2DM faced an increased risk of all-cause mortality and had a reduced life expectancy compared to the general population (29), which was consistent with our result. In the study by Moran C, et al., they reported that T2DM led to cognitive decline via neurodegeneration (30). In this study, a weighted logistic regression analysis adjusted for all confounders, revealed that individuals with T2DM had a 14% increased risk of cognitive impairment compared to those without T2DM. T2DM was found to be associated with cognitive impairment among older individuals. Also, the findings of the dispersion model suggested an inverse relationship between DSST scores and blood glucose levels. In simpler terms, hyperglycemia (HG) may be linked to cognitive impairment among the older adults. There was no doubt that our study was consistent with previous research. Cox DJ et al. highlighted that approximately 50% of individuals with diabetes developed HG-related cognitive impairment (31). The mechanism may be explained by insulin resistance, oxidative stress, chronic inflammation, arteriosclerosis, and mitochondrial dysfunction (32). Prior research has indicated that diabetes was risk factor for frailty among older adults, which can result in cognitive impairment (26, 33, 34). A connection was suggested by Mone P, et al., regarding the association between decline in physical health and cognitive function among elderly individuals with diabetes (26). However, since the inclusion of participants from the NHANES database, we lacked information on frailty in participants (such as the 5-m gait speed test). Consequently, it was not possible for us to conduct a subgroup analysis specifically targeting individuals who are considered frail.

Importantly, a mediation effect of cognitive impairment in the link between T2DM and all-cause mortality was observed in this analysis. To the best of our understanding, this study is believed to be the initial investigation examining the mediating role of cognitive impairment in the link between T2DM and all-cause mortality among US older adults. Our study also shown that there was no observed association between the coexistence of cognitive impairment and T2DM with regards to all-cause mortality. A previous study found a multiplicative negative interaction between diabetes mellitus and cognitive impairment regarding all-cause mortality (35), which was inconsistent with our findings. Li et al., used data form Beijing Elderly Comprehensive Health Cohort Study (BECHCS) which included both urban and rural areas of elderly Chinese individuals. The researchers employed the Chinese version of Mini-Mental State Examination score to evaluate cognitive function (35). However, our study exclusively included participants from NHANES, a stratified and multistage probability survey conducted on the population of the United States. We adopted DSST to evaluate cognitive function among older adults. Therefore, we guess that the observed variations may be attributed to disparities in participants selection, adjustment for confounding variables, and variations in the cognitive impairment assessment scale. More research is needed to explore this interaction between diabetes mellitus and cognitive impairment in relation to all-cause mortality. The percentage of mediation effect could be calculated according to the comparison of total effects with controlled direct effects. Our findings indicated that the mediation effect ratio of cognitive impairment was 16.2%, suggesting that cognitive impairment mediated 16.2% of the mediation effect on the all-cause mortality caused by T2DM. T2DM was not only directly related to all-cause mortality but also indirectly affected all-cause mortality through cognitive impairment. This suggested that all-cause mortality in patients with T2DM was partly due to their higher cognitive impairment. Some evidence and studies have indicated that individuals diagnosed with T2DM may face an increased risk of experiencing cognitive impairment compared to those without T2DM (36, 37). Petermann F et al., discovered that elderly individuals with diabetes were more likely to develop cognitive impairment and thus dementia (37). The underlying mechanism may be related to inflammation, hyperglycemia, the blood-brain barrier, and insulin resistance (38). Although the exact mechanism remained unclear, these explanations may support our finding that cognitive impairment mediated the link of T2DM with all-cause mortality.

It was worth mentioning that this finding provides support for the significance of preventing cognitive decline in elderly individuals with T2DM, potentially leading to a decrease in all-cause mortality. Nevertheless, it is important to acknowledge the limitations of our study. Firstly, the information regarding disease history such as CVD, hypertension, and dyslipidemia, was obtained through self-reporting by individuals from the NHANES database, which may be subject to recall bias. Secondly, DSST was employed for the evaluation of cognitive impairment. Currently, validated tests, Mini Mental State Examination (MMSE), and Montreal Cognitive Assessment (MoCA) are commonly employed to appraise global cognitive function (26, 39–41). However, the NHANES database did not include information on MMSE and MoCA. Further investigation is required to explore the potential impact of additional cognitive function tests on both all-cause mortality and T2DM. Additionally, some of the factors considered as confounders may themselves also be mediators, like history of CVD. Thirdly, this study collected data from NHANES database, we cannot determine a causal relationship of T2DM with cognitive impairment. It should be noted that NHANES gathers nationally representative data from the American older population, thereby limiting the generalizability of its findings to other countries. Fourthly, NHANES do not collect information regarding disease progression, lifestyle changes, which may be potential covariates. Finally, cognitive impairment and other covariates evaluated in this study were obtained at baseline, and changes during follow-up may have influenced results. Despite the limitations of the present investigation, our findings from this nationally representative database suggested a mediating role of cognitive impairment in the link of T2DM with all-cause mortality among older populations, which may provide a reference for actively screening for cognitive impairment among older adults with T2DM to help reduce the risk of mortality.

## Conclusion

This research discovered a mediating role of cognitive impairment in the link of T2DM with mortality among older people.

## Data availability statement

The original contributions presented in the study are included in the article/**Supplementary Material**. Further inquiries can be directed to the corresponding author.

## Ethics statement

Ethical review and approval was not required for the study on human participants in accordance with the local legislation and institutional requirements. Written informed consent from the patients/participants or patients/participants’ legal guardian/next of kin was not required to participate in this study in accordance with the national legislation and the institutional requirements.

## Author contributions

BW: Writing – original draft. JH: Writing – review & editing.

## Supplementary material

Supplementary Figure 1 Dispersion model between DSST and fasting glucose (r=-0.18, P<0.001).

Supplementary Figure 2 Dispersion model between DSST and HbA1c (r= -0.19, P<0.001).
