# Supplementary figures and images for: Mediation effect of cognitive impairment for the relationship of type 2 diabetes mellitus with mortality among elderly individuals
Source: Front Endocrinol (Lausanne). 2024 Jun 3;15:1392326. doi: 10.3389/fendo.2024.1392326 (PMC11180905; doi:10.3389/fendo.2024.1392326)

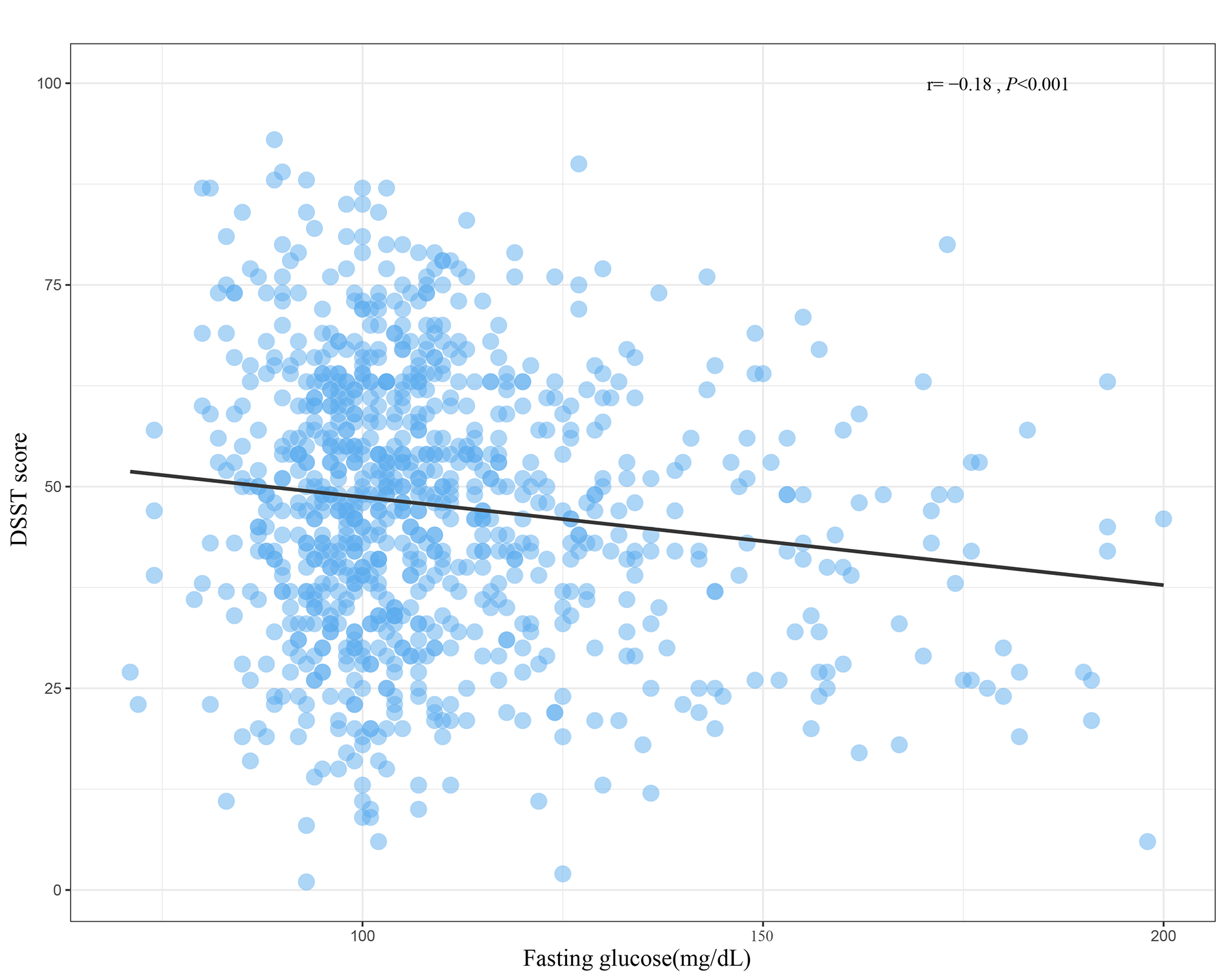

Supplement: Supplementary Figure 1 — Dispersion model between DSST and fasting glucose (r=-0.18, P<0.001). [file Image_1.tif]

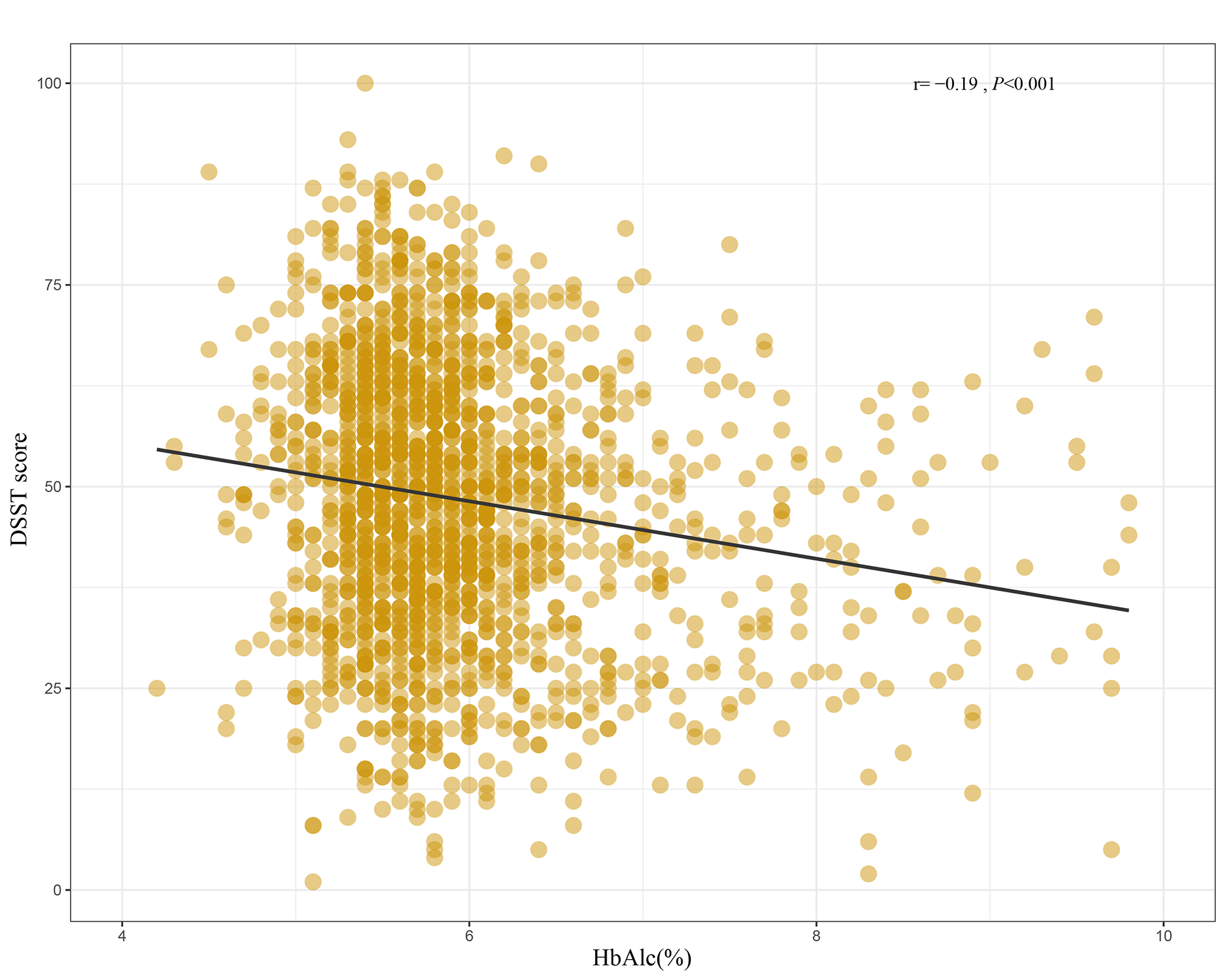

Supplement: Supplementary Figure 2 — Dispersion model between DSST and HbA1c (r= -0.19, P<0.001). [file Image_2.tif]
