# Supplementary material for: Mediation effect of cognitive impairment for the relationship of type 2 diabetes mellitus with mortality among elderly individuals
Source: Front Endocrinol (Lausanne). 2024 Jun 3;15:1392326. doi: 10.3389/fendo.2024.1392326 (PMC11180905; doi:10.3389/fendo.2024.1392326)
Supplement: Supplementary file 3 [file DataSheet_1.docx]

**Supplemental Table 1 The sensitivity analysis of missing variables.**

| **Variables** | **Number and proportion of missing variables, n (%)** | **Before interpolation** | **After interpolation** | **Statistics** | ***P*** |
| --- | --- | --- | --- | --- | --- |
| Marital status, n (%) | 4 (0.21%) |  |  | χ^2^=7.22 | 0.205 |
| Married |  | 1075 (64.56) | 1077 (64.55) |  |  |
| Widowed |  | 316 (14.43) | 316 (14.42) |  |  |
| Divorced |  | 283 (13.64) | 285 (13.66) |  |  |
| Separated |  | 52 (1.00) | 52 (1.00) |  |  |
| Never married |  | 105 (3.94) | 105 (3.94) |  |  |
| Living with partner |  | 56 (2.44) | 56 (2.44) |  |  |
| PIR, Mean (S.E) | 153 (8.09%) | 3.28 (0.09) | 3.26 (0.08) | t=1.33 | 0.193 |
| Smoking, n (%) | 2 (0.11%) |  |  | χ^2^=2.09 | 0.149 |
| No |  | 934 (49.47) | 936 (49.49) |  |  |
| Yes |  | 955 (50.53) | 955 (50.51) |  |  |
| Creatinine, Mean (S.E) | 101 (5.34%) | 0.96 (0.01) | 0.96 (0.01) | t=0.24 | 0.815 |
| Cancer | 2 (0.11%) |  |  | χ^2^=1.98 | 0.159 |
| No |  | 1513 (76.07) | 1515 (76.08) |  |  |
| Yes |  | 376 (23.93) | 376 (23.92) |  |  |

PIR= poverty-to-income ratio.
