# Supplementary material for: Mediation effect of cognitive impairment for the relationship of type 2 diabetes mellitus with mortality among elderly individuals
Source: Front Endocrinol (Lausanne). 2024 Jun 3;15:1392326. doi: 10.3389/fendo.2024.1392326 (PMC11180905; doi:10.3389/fendo.2024.1392326)
Supplement: Supplementary file 4 [file DataSheet_2.docx]

**Supplemental Table 2 The possible confounding factors associated with all-cause mortality by a weighted univariate COX analysis.**

| Variables | HR (95%CI) | *P* |
| --- | --- | --- |
| Age | 1.16 (1.13-1.20) | <0.001 |
| Gender |  |  |
| Male | Ref |  |
| Female | 0.70 (0.52-0.94) | 0.019 |
| Race |  |  |
| Non-Hispanic White | Ref |  |
| Non-Hispanic Black | 0.94 (0.65-1.36) | 0.735 |
| Other | 0.64 (0.40-1.02) | 0.061 |
| Marital status |  |  |
| Married | Ref |  |
| Never married | 1.64 (0.89-3.04) | 0.112 |
| Other | 1.63 (1.13-2.35) | 0.011 |
| BMI | 0.98 (0.96-1.01) | 0.192 |
| PIR | 0.79 (0.70-0.90) | <0.001 |
| Smoking |  |  |
| No | Ref |  |
| Yes | 1.48 (1.10-1.99) | 0.012 |
| Drinking alcohol |  |  |
| No | Ref |  |
| Yes | 0.90 (0.61-1.33) | 0.593 |
| Physical activity |  |  |
| > 360 MET·min | Ref |  |
| $\leq$360 MET·min | 1.54 (1.13-2.09) | 0.007 |
| History of CVD |  |  |
| No | Ref |  |
| Yes | 2.16 (1.48-3.17) | <0.001 |
| History of hypertension |  |  |
| No | Ref |  |
| Yes | 1.98 (1.34-2.93) | 0.001 |
| History of dyslipidemia |  |  |
| No | Ref |  |
| Yes | 0.64 (0.37-1.10) | 0.106 |
| History of cancer |  |  |
| No | Ref |  |
| Yes | 1.51 (1.03-2.21) | 0.037 |
| eGFR | 0.97 (0.96-0.98) | <0.001 |
| MED score | 0.99 (0.91-1.07) | 0.723 |
| Depression |  |  |
| PHQ-9 scores < 10 | Ref |  |
| PHQ-9 scores ≥ 10 | 0.84 (0.46-1.54) | 0.563 |
| T2DM |  |  |
| No | Ref |  |
| Yes | 1.45 (1.10-1.93) | 0.011 |
| DSST score |  |  |
| DSST score > 36 | Ref |  |
| DSST score ≤ 36 | 2.63 (1.78-3.89) | <0.001 |

BMI=body mass index; PIR=poverty-to-income ratio; CVD=cardiovascular disease; eGFR=estimate glomerular filtration rate; MED=Mediterranean Diet; MET=metabolic equivalent; PHQ-9=Patient Health Questionnaire-9; T2DM=type 2 diabetes mellitus; DSST=digit symbol substitution test; HR=hazard ratio; CI=confident interval.
